# Introducing effective genes in lymph node metastasis of breast cancer patients using SHAP values based on the mRNA expression data

**DOI:** 10.1371/journal.pone.0308531

**Published:** 2024-08-16

**Authors:** Sepideh Zununi Vahed, Seyed Mahdi Hosseiniyan Khatibi, Yalda Rahbar Saadat, Manijeh Emdadi, Bahareh Khodaei, Mohammad Matin Alishani, Farnaz Boostani, Solmaz Maleki Dizaj, Saeed Pirmoradi

**Affiliations:** 1 Kidney Research Center, Tabriz University of Medical Sciences, Tabriz, Iran; 2 Rahat Breath and Sleep Research Center, Tabriz University of Medical Science, Tabriz, Iran; 3 Department of Computer Engineering, Abadan Branch, Islamic Azad University, Abadan, Iran; 4 Clinical Research Development Unit of Tabriz Valiasr Hospital, Tabriz University of Medical Sciences, Tabriz, Iran; 5 Department of Computer Science, Faculty of Information Technology, University of Shahid Madani of Tabriz, Tabriz, Iran; 6 Dental and Periodontal Research Center, Tabriz University of Medical Sciences, Tabriz, Iran; State University of New York at Oswego, UNITED STATES OF AMERICA

## Abstract

**Objective:**

Breast cancer, a global concern predominantly impacting women, poses a significant threat when not identified early. While survival rates for breast cancer patients are typically favorable, the emergence of regional metastases markedly diminishes survival prospects. Detecting metastases and comprehending their molecular underpinnings are crucial for tailoring effective treatments and improving patient survival outcomes.

**Methods:**

Various artificial intelligence methods and techniques were employed in this study to achieve accurate outcomes. Initially, the data was organized and underwent hold-out cross-validation, data cleaning, and normalization. Subsequently, feature selection was conducted using ANOVA and binary Particle Swarm Optimization (PSO). During the analysis phase, the discriminative power of the selected features was evaluated using machine learning classification algorithms. Finally, the selected features were considered, and the SHAP algorithm was utilized to identify the most significant features for enhancing the decoding of dominant molecular mechanisms in lymph node metastases.

**Results:**

In this study, five main steps were followed for the analysis of mRNA expression data: reading, preprocessing, feature selection, classification, and SHAP algorithm. The RF classifier utilized the candidate mRNAs to differentiate between negative and positive categories with an accuracy of 61% and an AUC of 0.6. During the SHAP process, intriguing relationships between the selected mRNAs and positive/negative lymph node status were discovered. The results indicate that GDF5, BAHCC1, LCN2, FGF14-AS2, and IDH2 are among the top five most impactful mRNAs based on their SHAP values.

**Conclusion:**

The prominent identified mRNAs including GDF5, BAHCC1, LCN2, FGF14-AS2, and IDH2, are implicated in lymph node metastasis. This study holds promise in elucidating a thorough insight into key candidate genes that could significantly impact the early detection and tailored therapeutic strategies for lymph node metastasis in patients with breast cancer.

## Introduction

Breast cancer is a widespread and fatal form of cancer that affects women globally [1]. In 2023, 297,790 new cases of breast cancer and 43,700 deaths were reported in the US [2]. The survival rate for breast cancer patients is high, with 90% and 83% at 5 and 10 years, respectively [3]. However, the presence of metastases in breast cancer patients leads to a notable decrease in survival rates. In cases where there are regional metastases, such as in the lymph nodes, the 5-year survival rate drops to 85%. If the metastases are distant, the rate decreases further to 26% [4]. Identifying the presence of metastases is of utmost importance as it allows for appropriate treatment and ultimately enhances patient survival rates.

When looking for the presence of metastases, the first step is to examine the regional lymph nodes. The presence of metastases in these lymph nodes is a poor prognostic factor only, while it is the main factor in predicting the presence of distant metastases [5]. In the case of breast cancer, the most common method of evaluating the regional lymph node status is the sentinel lymph node procedure [6, 7]. This procedure involves injecting a blue dye or radioactive tracer near the tumor. The first lymph node reached by the injected substance, known as the sentinel lymph node, is likely to contain metastatic cancer cells and should be removed. The removed lymph node is then sent for pathological processing and analysis by a pathologist.

Evaluating lymph node status is a crucial task for pathologists, but it comes with a challenge. The large area of tissue that needs to be checked is extensive, and it can be hard to identify metastases that can be as small as single cells. In the case of sentinel lymph nodes, at least three sections at different levels through the lymph node have to be examined. At least ten lymph nodes of non-sentinel nodes must be examined [8, 9]. This process can be tedious and time-consuming, and pathologists may miss small metastases [10]. To address this, pathologists in the Netherlands performed a secondary examination using immune histochemical staining for cytokeratin if inspection of the H&E slide reveals no metastases. However, even in this secondary examination, metastases can still be missed [11].

With the advent of personalized treatment, traditional prognostic biomarkers such as tumor size, tumor grade, and lymph node metastases are no longer sufficient for the effective management of early-diagnosed breast cancer patients [12, 13]. As a result, extensive research has been conducted to identify and validate molecular biomarkers in recent years that can serve as prognostic and predictive indicators. These new approaches are commonly referred to as multi-parameter, multi-analyte, and multi-gene tests. Several of these tests, recommended by experts, are currently used in clinical practice. Some validated examinations include Oncotype DX, MammaPrint, and uPA/PAI-1. The Oncotype DX test is a widely used multigene signature test that helps predict the risk of breast cancer recurrence by evaluating the expression of 21 mRNA genes and then calculates a recurrence score based on the relative expression of these genes [14, 15]. However, it should be noted that Oncotype DX has some limitations, such as a lack of validation for long-term follow-ups and ER-negative cases. Another molecular test called MammaPrint uses micro-array to evaluate the relative expression of genes associated with regulatory pathways of cancer, specifically 70 genes. MammaPrint is a validated test for predicting cancer recurrence and dividing patients into high-risk and low-risk groups [16–20]. In addition to these tests, there is another molecular test that evaluates protein levels. This test measures uPA and PAI-1 markers by extracting breast cancer tissues [21]. Studies have shown that elevated levels of these proteins result in more severe outcomes in patients.

These multigene signature tests are exorbitant in many countries. To set up a simple and inexpensive test to serve as a diagnostic and predictive biomarker test, considerable effort has been devoted. To discover a novel biomarker, databases could be considered a helpful tool as well. Recently, many researchers have been working on circulating tumor cells (CTCs), microRNAs, and DNA mutation testing (such as the measurement of ctDNA) to find new prognostic and predictive markers. These novel biomarkers before their clinical applications should be validated through clinical and analytical assessments to start our journey towards a personalized treatment for early-diagnosed patients with breast cancer, we need established prognostic biomarkers in combination with validated prognostic/predictive factors.

In this study, we aimed to create a new roadmap for the development of robust biomarkers that can accurately detect the presence of lymph node metastases. The significance of this work lies in its ability to provide effective pathways for identifying patients who may have complex conditions where traditional clinical and imaging diagnostic methods fail to detect metastases despite the presence of metastases from a pathological perspective. Our research has introduced new perspectives and tools to assist pathologists and surgeons in screening and classifying such complex and ambiguous conditions and to facilitate decision-making when considering the possible removal of axillary lymph nodes.

## Material and methods

### Material

The NCBI data portal (https://www.ncbi.nlm.nih.gov/geo/) provides mRNA-seq data from the GEO repository. For this study, we used dataset number GSE96058, which contains 30,865 gene expressions (mRNAs) for 3409 breast cancer patients measured using the GPL11154 platform [22, 23]. This dataset is a subset of the multi-center prospective cohort study Sweden Cancerome Analysis Network—Breast [SCAN-B], in which gene expression data is collected from multiple clinical centers. This platform applied the Illumina HiSeq 2000 technique, in which gene expression levels are reported using 54,715 Probes for 30,865 genes. In addition, clinical data of breast cancer patients were obtained from the same dataset. The breast cancer patients were divided into two groups based on their lymph node status: 2099 samples were in the negative lymph node group, while 1209 were in the positive lymph node group (Table 1). Informed consent to participate was obtained from all patients by the Regional Ethical Review Board of Lund (diary numbers 2007/155, 2009/658, 2009/659, 2014/8), the county governmental biobank center, and the Swedish Data Inspection group (diary number 364–2010).

**Table 1:** GSE96058 information.

GSE96058 (Lymph Node Status)		
Positive	Negative	Not reported
1209	2099	110

The study was conducted by the principles of the Declaration of Helsinki (2013), and we received permission to access the research data file from the NCBI-GEO program through the National Cancer Institute in the United States. Since the NCBI-GEO data is publicly available, the local ethics committee waived the need for approval. The local ethics code is IR.NIMAD.REC.1400.025.

## Method

**Fig 1:**
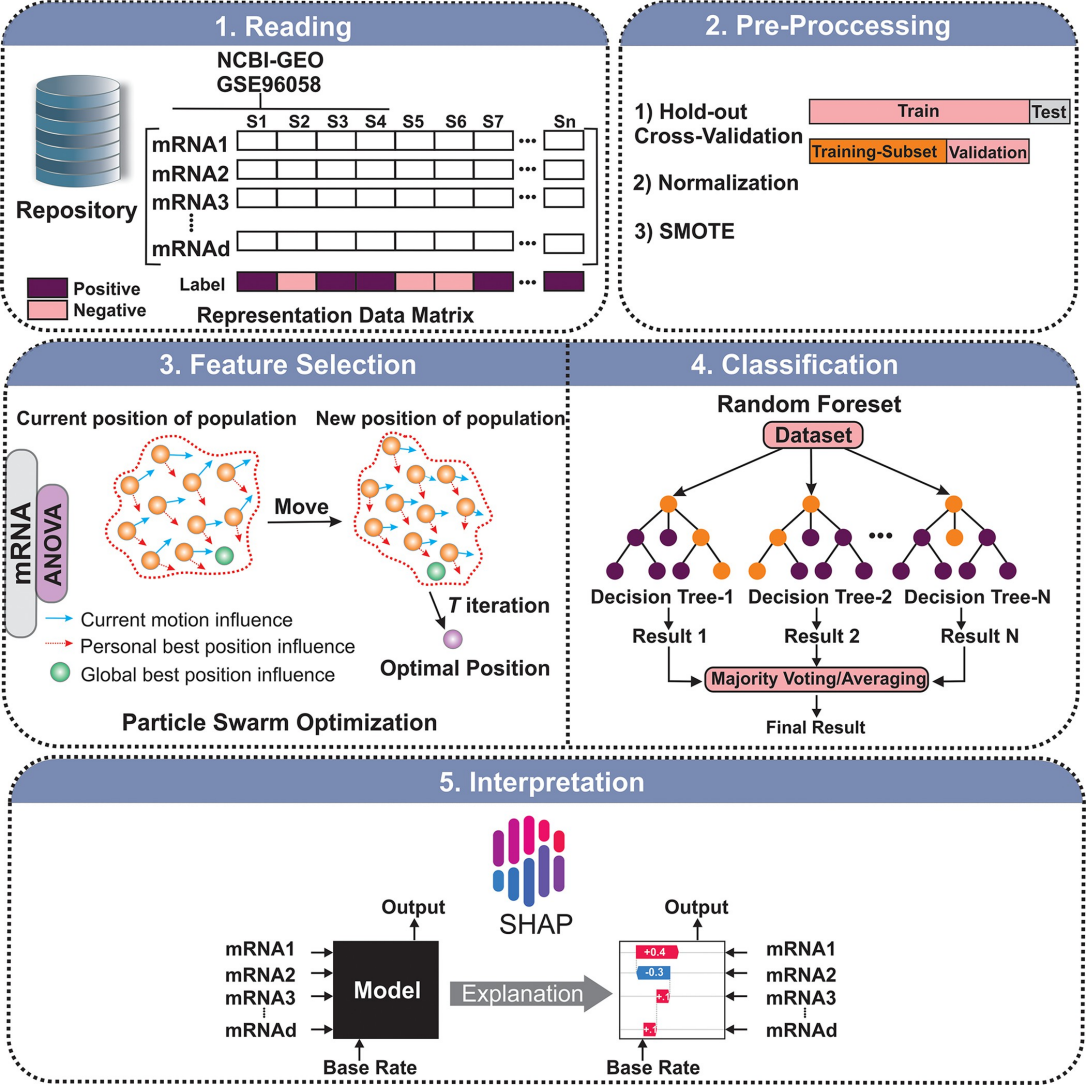
The overview of the proposed method. Five main steps, including reading, preprocessing, feature selection, classification, and SHAP algorithm were applied to the mRNA expression data. 1) Required data was collected from the NCBI-GEO repository and organized during the reading step. 2) The pre-processing step includes two sub-steps, cross-validation and data normalization. 3) The feature-selection step contains two parts: the filter method based on ANOVA and the wrapper method based on Particle Swarm Optimization (PSO) for mRNA data, in which candidate mRNAs with more relevance to positive and negative Papillary lymph node groups were selected. 4) Multi-classifier models were utilized to evaluate the discrimination power of the selected mRNAs. 5) The SHAP algorithm was employed to discover the possible relationship between the selected mRNAs and positive and negative groups.

The proposed approach for finding significant mRNAs involves five steps: reading, pre-processing, feature selection, classification, and applying the SHAP algorithm. Fig 1 illustrates the complete process and provides additional details for each step.

In the reading step, the mRNA data was organized into a matrix with 3308 rows and 30,865 columns, representing the number of samples and features respectively. To achieve a more authentic error estimation, the hold-out Cross-Validation (CV) approach was used to separate the data into train, validation, and test proportions. The train, validation, and test sets were set to 70%, 10%, and 20%, respectively. Additionally, some feature columns with identical values for all samples of the training set were removed. Finally, to normalize the feature selection and classification steps, the z-score and min-max methods were employed.

To reduce the number of irrelevant attributes, we implemented a two-part feature-selection process that consisted of a classifier-independent filter method and a wrapper method [24]. During the filter phase, we used ANOVA [25] to evaluate each feature individually and reduce the dimension of the mRNA data. Due to class imbalance in the training set, we used the SMOTE algorithm [26] to reduce its effect on ANOVA. This also helped to reduce the computational cost during the subsequent wrapper step. From the training set, we selected the 200 top features based on their F-values.

We used a method called Particle Swarm Optimization (PSO) [27] to select the most important features. This method is based on swarm intelligence [28] and requires a classifier, in our case Random Forest (RF), to evaluate the fitness function. To ensure accuracy, we chose the fitness function based on AUC and measured its value using a validation set. The algorithm parameters, including the number of population and iterations, were set to 35 and 100 respectively. After running the binary PSO method, we selected 109 significant mRNAs based on the output.

Different machine learning models are not inherently better than others, as each model can outperform the others depending on the problem at hand. Therefore, it is crucial to employ various methods to address the issue, particularly in the classification domain. Subsequently, the best-performing model, free from bias, is selected based on its performance. To determine the most suitable model, various parameters are taken into consideration, with the AUC being one of the most valuable parameters according to machine learning literature. We used several supervised classifiers, such as Support Vector Machine (SVM) [29], Naive Bayes (NB), K-Nearest Neighbor (KNN) [30], and Random Forest (RF) [31], to determine the differentiation power of the selected mRNAs. For each classifier, important evaluation metrics including accuracy and AUC were calculated.

In the next step, we studied significant relationships using the SHapley Additive exPlanation (SHAP) algorithm [32]. We extracted SHAP values regarding the effect of selected mRNAs in model predictions (positive/negative lymph node status). It is crucial to accurately understand the output of a prediction model. This helps build user trust, identify areas for improvement, and gain insights into the modelling process. Simple models, such as linear models, are often preferred in certain applications due to their ease of interpretation, even if they may not be as accurate as complex models. However, the increasing availability of big data has highlighted the trade-off between a model’s accuracy and interpretability. Various methods have been proposed to address this issue, but there is still a lack of understanding about how these methods compare and when to use one over the other.

The SHAP algorithm is an effective tool for explaining the role of each input variable in model predictions. The SHAP technique employs simplified explanation models that yield close approximations to the original predictive model. These models help in explaining complex machine learning models.

The detailed technical explanation for each of the stages mentioned in the methodology has been comprehensively covered in the original references. Additionally, the output results of each step have been reported in the results section of the study. It is worth mentioning that Python was the primary programming language used in this study, and we employed various libraries and frameworks such as Numpy, Pandas, Matplotlib, Seaborn, Scikit-learn, Scipy, Pyswarms, and SHAP to implement the proposed steps.

## Result

The current study had two primary objectives. The primary aim was to decipher the molecular mechanisms implicated in lymph node metastasis and isolate the top mRNAs with the most associative interactions. The secondary objective was to identify the most significant mRNAs capable of accurately distinguishing positive and negative lymph node status in breast cancer. Table 2 reports the top 109 most significant features among the 28,456 mRNAs. After two consecutive feature selection steps, these features were selected using ANOVA (filter method) and binary PSO algorithm (wrapper method).

**Table 2:** The top 109 most significant features among the 28,456 mRNAs.

#	Gene Name	#	Gene Name	#	Gene Name	#	Gene Name
1	DLGAP1-AS5	31	AK023627	61	ERRFI1	91	LOC256880
2	HOXC10	32	MYOC	62	FOS	92	LOC100130899
3	HOXC13	33	FGF14-AS2	63	ARRDC1	93	HGD
4	PQLC3	34	USP13	64	IQCB1	94	ORMDL2
5	PSCA	35	BAHCC1	65	HMOX2	95	TMEM40
6	NRBP1	36	MMP11	66	LOC102577424	96	RAMP2-AS1
7	PHLDA1	37	DQ595103	67	MPL	97	QPRT
8	SDC1	38	GDPD3	68	BRE-AS1	98	LCN2
9	AVEN	39	SMARCA1	69	RAPGEF4	99	PPP4C
10	CUEDC1	40	LOC730101	70	RAC1	100	LMOD2
11	NFYC-AS1	41	ZNF702P	71	TM4SF1-AS1	101	STIM1
12	TM4SF18	42	PC	72	SLC35C1	102	PPAPDC1A
13	CITED2	43	SLC38A4	73	ABHD11	103	PPP6R2
14	S100P	44	ARMC7	74	SAMD11	104	UNC119B
15	SHC3	45	LOC100506548	75	PLXNC1	105	SEPHS2
16	CNKSR3	46	AF086258	76	RAMP1	106	TTC17
17	TMEM150A	47	TFPI2	77	GRPR	107	STK17A
18	IRS2	48	NFIX	78	HOXC12	108	AQP6
19	CACNB2	49	HSPB1	79	STRA13	109	NCOA3
20	HOPX	50	ZNF529	80	DERL3		
21	ADRA1B	51	PAX9	81	LINC00899		
22	C4orf19	52	PEX5	82	SLC38A5		
23	C7orf34	53	MYC	83	ETNK2		
24	GDF5	54	CCDC91	84	DPY19L2		
25	LOC100506860	55	PTK6	85	ACAN		
26	ATN1	56	MIRLET7DHG	86	HARS2		
27	KLHL15	57	AK127179	87	NCCRP1		
28	DYRK2	58	ZNF815P	88	ACOT6		
29	ACTB	59	CLU	89	SPDEF		
30	ZFAND5	60	MFI2-AS1	90	IDH2		

To assess the effectiveness of the selected mRNAs in distinguishing between positive and negative lymph nodes, four different classifiers, namely SVM, KNN, NB, and RF were used. The performance of each classifier was evaluated by computing key metrics such as accuracy and AUC across the train/validation/test folds of the candidate mRNAs and reported in Table 3.

**Table 3:** The performance of classifiers.

Classifier	Training set		Test set	
	Accuracy (%)	AUC	Accuracy (%)	AUC
RF	70	0.7	61	0.6
SVM (RBF Kernel)	83	0.78	65	0.56
KNN	73	0.7	61	0.55
NB	62	0.62	58	0.58

**Fig 2:**
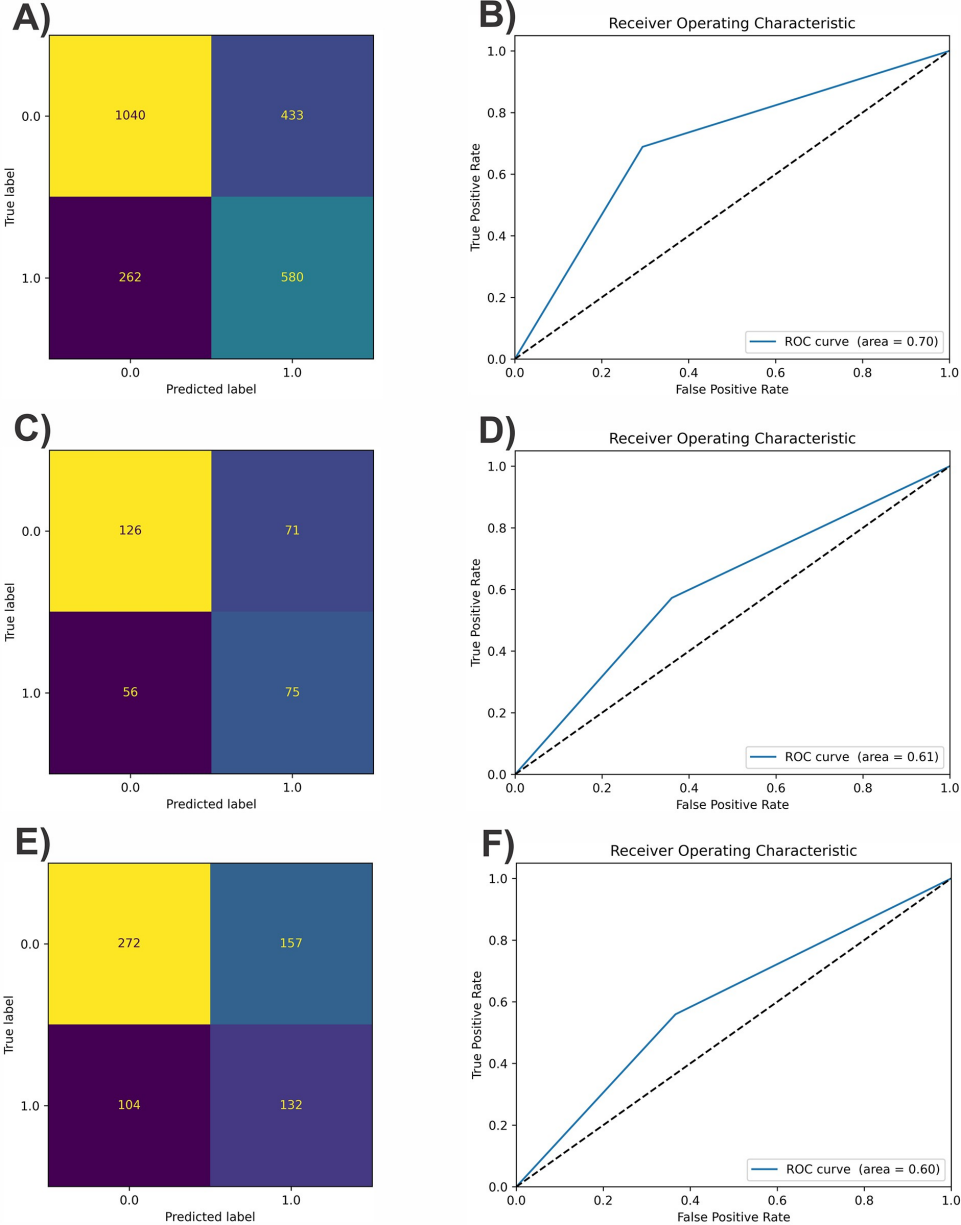
The confusion matrix for (a): Training set Confusion Matrix, (b): Training set ROC (c): Validation set Confusion Matrix, (d): Validation set ROC (e): Test set Confusion Matrix, (f): Test set ROC. Zero and one are negative and positive groups, respectively.

The classifiers rely on mRNA features and the results showed that RF outperforms other algorithms. The accuracy of RF was 61%, and its AUC-ROC score was 0.6 in the test data evaluation. Fig 2 illustrates the confusion matrices for the training, validation, and test sets.

**Fig 3:**
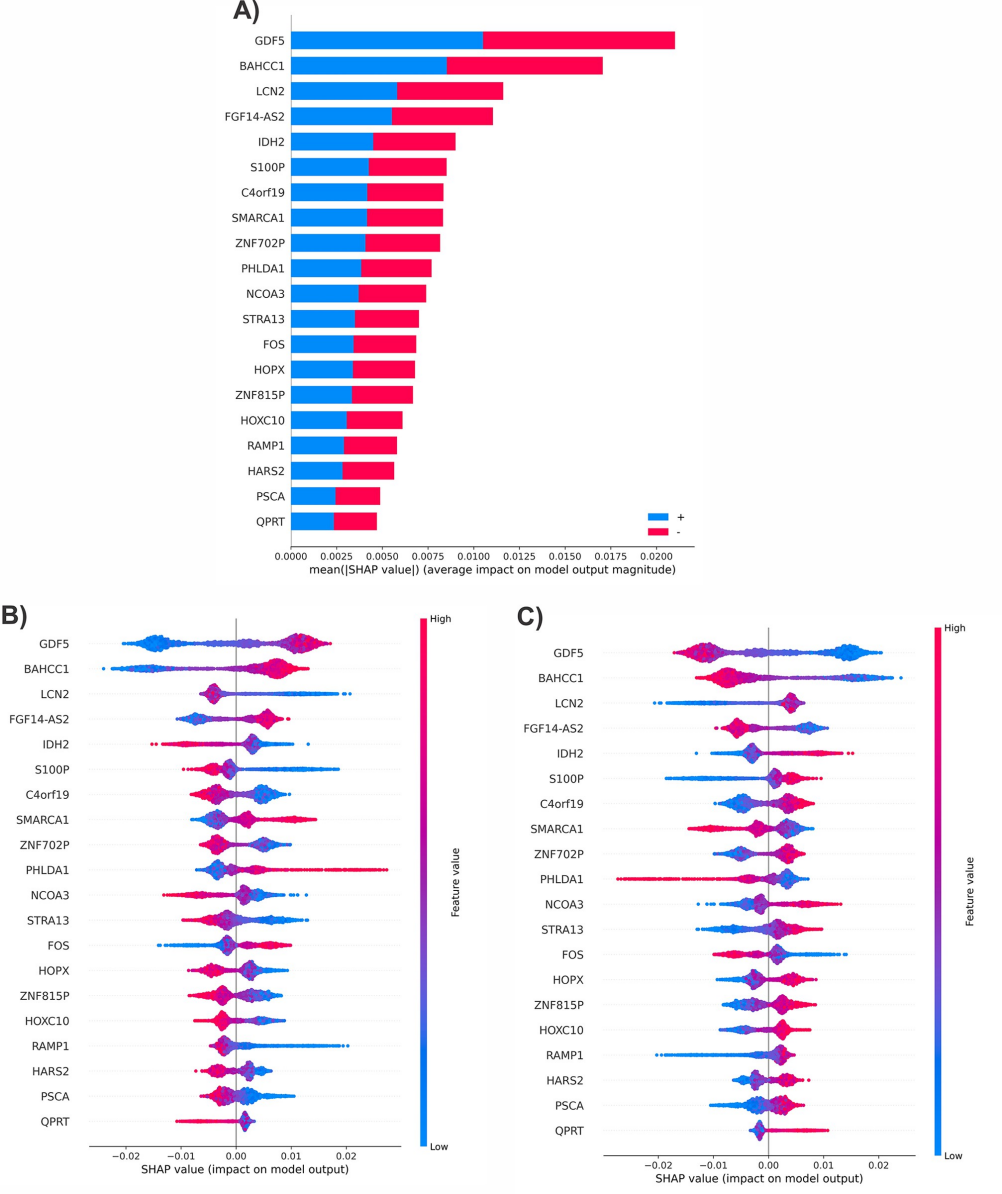
The summary plot using SHAP values (a): summary plot of both classes, (b): summary plot of the negative group, (c): summary plot of the positive group.

During the process of SHAP, some interesting relationships between selected mRNAs and positive/negative lymph node status were discovered. Additionally, the five most significant mRNAs based on their SHAP values were selected and their role in the positive and negative lymph nodes of breast cancer patients were studied to gain a better understanding of their impact and relevance. In this study, a summary plot is presented using SHAP values, displayed in Fig 3A. This plot showcases the significance and impact of selected mRNAs in model predictions for both negative and positive lymph nodes. The results indicate that GDF5, BAHCC1, LCN2, FGF14-AS2, and IDH2 are among the top five most effective mRNAs based on their SHAP values. Additionally, two separate summary plots are provided based on negative and positive lymph node classes, shown in Fig 3B and 3C, respectively. The Y-axis in these plots lists feature names in the order of their importance, from top to bottom, while the X-axis represents the SHAP value, indicating the degree of change in log odds. The color of each point on the plot represents the value of the corresponding mRNA, where red indicates high values and blue indicates low values. Each point in the plot represents a row of mRNA expression data from the original dataset. If we observe the SHAP value of GDF5, we can see that it is mostly negative when the count of GDF5 is low. This indicates that lower expression of GDF5 counts tends to negatively impact the output.

**Fig 4:**
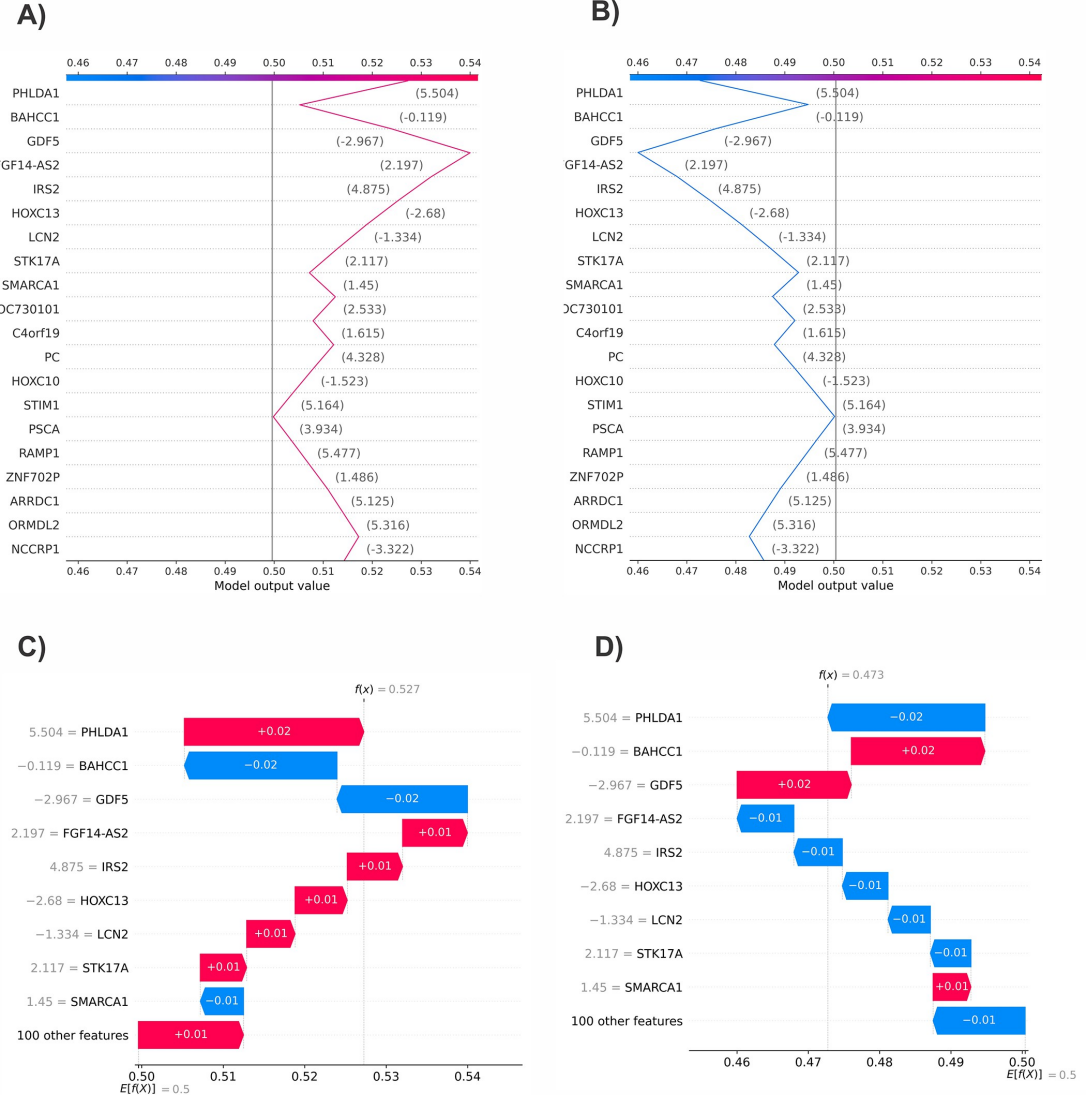
(a): Decision plot of the first sample related to negative class, (b): Decision plot of the first sample related to positive class, (c): waterfall plot of the first sample related to negative class, (d): waterfall plot of the first sample related to positive class.

We presented the decision plot, which illustrates the model’s decisions by displaying the cumulative SHAP values for each prediction or sample. The decision plot features lines that show the extent to which each feature contributed to a specific model prediction, thereby explaining which feature values influenced the prediction. In the decision plots of Fig 4A and 4B, the negative and positive target labels are respectively represented. The explanation in Fig 4C (negative class) and d (positive class) displays the features that contribute to the model prediction, compared to the average model output over the training dataset. The features that push the prediction higher are shown in red, while those that push it lower are shown in blue. This explanation is presented in a waterfall plot format.

**Fig 5:**
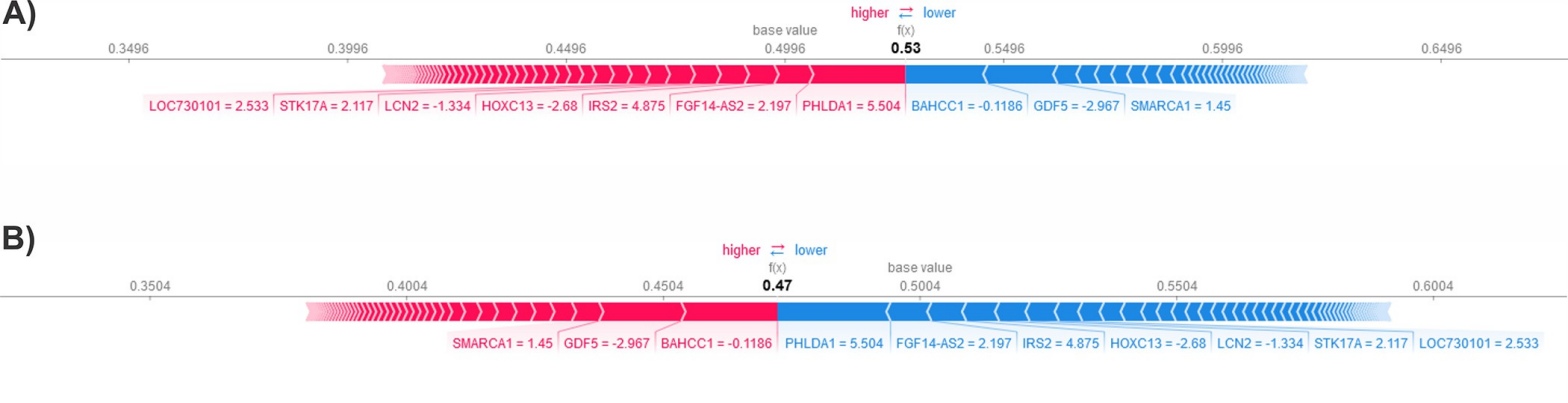
The force plot of the first sample in dataset (a): Force plot of negative class, (b): Force plot of positive class.

**Fig 6:**
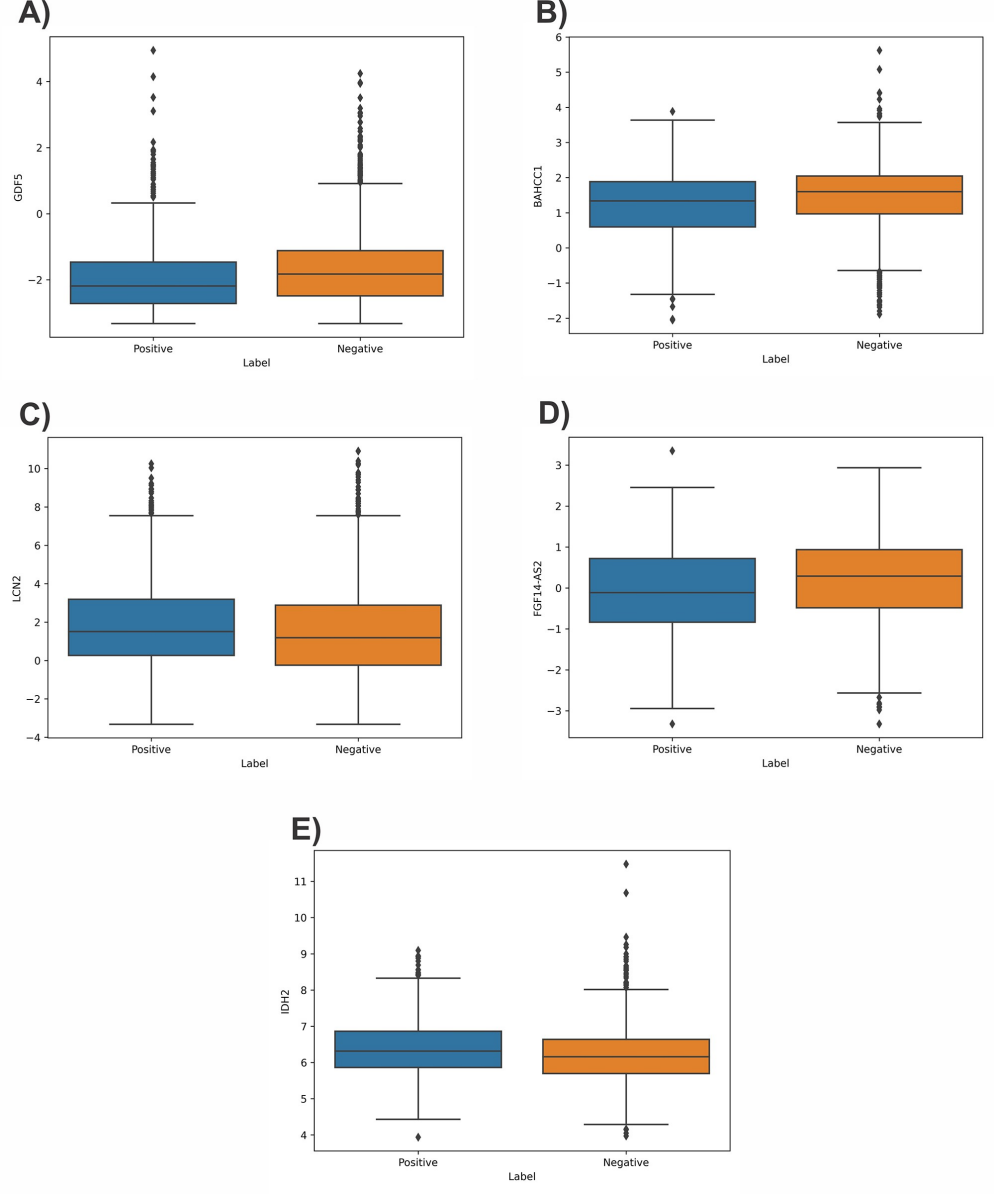
The box plot of five more important mRNAs based on their SHAP values (a): GDF5, (b): BAHCC1, (c): LCN2, (d): FGF14-AS2, (e): IDH2.

The first sample in the dataset was analyzed to identify the features that led to positive and negative outcomes (Fig 5A and 5B). A force plot was used to provide the expected value, SHAP value, and testing sample. From the negative class in Fig 5A, it was found that PHLDA1 and FGF14-AS2 increased the results, while BAHCC1 and GDF5 decreased them. Fig 5B also provided other details related to the positive class. Fig 6 displays the box plot of five critical mRNAs with the most significant effect on model predictions. These mRNAs were identified based on the SHAP values.

## Discussion

The presence of lymph node metastasis demonstrates a strong correlation with the recurrence of cancer after surgery and the length of time a patient survives post-surgery. Therefore, it is of utmost importance to identify biomarkers that can predict the early occurrence of lymph node metastasis in breast cancer. Our results show that GDF5, BAHCC1, LCN2, FGF14-AS2, and IDH2 are among the top five most effective mRNAs involved in lymph node metastasis of breast cancer based on their SHAP values.

Changes in the vasculature of the lymph nodes during malignant progression could create an environment conducive to the attraction and maintenance of tumor cells, thereby establishing a specialized niche for metastasis [33]. The GDF5 gene (also called BMP14; bone morphogenetic protein-14) was the first mRNA identified in this study. It is a member of the transforming growth factor ß (TGF-ß) superfamily [34]. In human vascular endothelial cells, GDF5 facilitates the proangiogenic action of vascular endothelial growth factor [35], and controls the up-regulation of urokinase-type plasminogen activator (uPA) receptor (uPAR), and plays a role in angiogenesis [36] by promoting the migration of aortic endothelial cells without affecting their proliferation [37]. The overproduction of TGF-ß in breast cancer cells elicits the expression of GDF5 in endothelial cells, promoting angiogenesis. The proangiogenic effect of GDF5 can be regulated by anti-TGF-ß peptides and anti-GDF5 antibodies and applied as potential therapeutic tools for TGF-ß/GDF5-dependent breast cancer angiogenesis [38]. Recently, GDF5 has been also found to be associated with lymph node metastasis in colorectal cancer [39]. The absence of GDF5 may have significant implications on the immune responses and the functionality of macrophages [40]. Based on the available reports, GDF5 can be a valuable molecular target for targeting the modulation of metastasis of lymph nodes in breast cancer.

Cancer cells experience extensive alterations in their transcriptional profile by the development of alternative gene regulatory elements like super-enhancers [41]. Based on the results of this study, the BAHCC1 ranked as the second significant mRNA in lymph node metastasis of breast cancer samples. The presence of BAHCC1, a super-enhancer, is crucial for the successful growth, engraftment, and dissemination of tumors. BAHCC1 acts as a transcriptional regulator, controlling the expression of DNA-repair and E2F/KLF-dependent cell-cycle genes. Silencing of BAHCC1 leads to reduced cell proliferation and delayed DNA repair [41]. Furthermore, it interacts with important transcriptional corepressors, namely histone deacetylase and SAP30-binding protein, thereby providing a molecular foundation for BAHCC1-mediated suppression of target genes. BAHCC1 is extensively overexpressed in various subtypes of human acute leukemia and plays a vital role in the growth of malignant cells in animals and cell lines. In leukemia, the depletion of BAHCC1 leads to the inhibition of oncogenesis [42]. Notably, BAHCC1 forms associations with BRG1-containing remodeling complexes at the promoters of these genes. The mechanism by which the BAHCC1 is involved in lymph node metastasis has not been defined in breast cancer and our results suggest elucidating its direct role in breast cancer.

Lipocalin-2 (LCN2), a secretory glycoprotein, was identified as a top mRNA with the highest role in metastasis of the lymph node. Lipocalin-2 is responsible for the transportation of small lipophilic ligands. Its role in breast cancer and the metastasis of lymph nodes is of utmost importance. Aberrant expression of LCN2 plays a crucial role in various processes related to breast cancer such as angiogenesis, invasion and migration of cells, and the transition of epithelial cells to mesenchymal cells (EMT). An increase in the expression of LCN2 is linked with a negative prognosis, the presence of lymph node metastasis, the grading of tumors, and the estrogen receptor (ER)- negative status [43]. Lipocalin-2 actively encourages the metastasis of breast cancer by inducing angiogenesis, the production of vascular endothelial growth factor (VEGF), EMT, and the migration and invasion of cells, all of which occur through various signaling pathways including the PI3K/AKT/NF-κB [44], HIF-1α/Erk, ERα/Slug axis [45], and stabilizing matrix metalloproteinase-9 [46]. Taking into consideration the aforementioned discoveries, it can be concluded that LCN2 stimulates the invasion and metastasis of breast cancer cells by inducing EMT and promoting angiogenesis. This leads to the suggestion that LCN2 could be a potential target for therapeutic interventions aimed at inhibiting the progression of breast cancer. Agents that are capable of reducing and preventing the secretion of LCN2 are expected to have a wide range of applications and be beneficial for patients who are suffering from breast cancer [44, 47–51].

The 4^th^ identified mRNA in this study was FGF14 antisense RNA 2 (FGF14-AS2), an emerging long non-coding RNA. FGF14-AS2 was originally identified as a suppressor of tumor formation in breast cancer. In comparison to its expression in adjacent normal tissue, FGF14-AS2 displays a notable decrease in breast cancer tissues. This reduced expression of FGF14-AS2 is closely linked to an enlargement in tumor size, an advanced clinical stage, a higher occurrence of lymph node metastasis, and an overall survival rate that is more unfavorable [52]. According to a study conducted by Jin et al., FGF14-AS2 functions as a competitive endogenous RNA (ceRNA) of miR-370-3p, thereby promoting the expression of FGF14 at the post-transcriptional level in breast cancer [53]. Furthermore, it has been reported that FGF14-AS2 directly binds to miR-205-5p, leading to the inhibition of proliferation, migration, invasion, and the initiation of apoptosis in breast cancer [54]. These studies support and validate the functional roles of the identified FGF14-AS2 in lymph node metastasis of breast cancer.

Isocitrate dehydrogenase or IDH serves as a vital enzyme with rate-limiting capabilities within the tricarboxylic acid cycle (TCA cycle), which plays a crucial role in energy metabolism. The TCA cycle upregulates cellular energy in cancer cells that are highly proliferative and have metastasized. The expression levels of IDH isoforms have been observed to be dysregulated in various human malignancies, suggesting their involvement in oncogenesis. In the context of breast cancer, upregulation or mutation of the mitochondrial NADP-dependent enzyme IDH2 has been associated with disease progression and prognostics. The presence of IDH2 has been linked to the aggressive behavior of breast cancer through its promotion of cell proliferation. Furthermore, the status of IDH2 has been identified as a robust predictor of outcome, particularly in individuals with ER-positive breast cancer [55]. The wild-type *IDH2* [56] and its elevated expression potentially play a critical function in the progression of breast cancer and the emergence of lymphovascular invasion and metastasis [57]. Notably, IDH2 was found to be significantly upregulated in stage 3 breast cancer tissues and cell lines. Its presence was shown to be essential for arresting the cell cycle and inducing breast cancer proliferation [58]. These findings along with our results confirm that IDH2 presents a promising target for the treatment of breast cancer.

Our study has some limitations. While our initial AI-based analysis focused on detecting significant genes associated with lymph node metastasis, a post-hoc pathway enrichment analysis did not be performed to provide additional biological insights into the potential mechanisms and signaling pathways involved in the metastatic process. Moreover, we have not validated the identified RNA/protein expression levels in clinical samples. Nevertheless, existing studies supports and reinforce the molecular mechanism and biological functions of the candidate RNAs in lymph node metastasis of breast cancer.

Apart from the 5 genes we identified, there are other identified genes that are previously reported to be related to lymph node metastases. Our literature review reveals that dysregulated levels of identified RNAs including PQLC3 [59], Syndecan-1 (SDC1; a heparin sulfate proteoglycan) [60, 61], IRS2 (Insulin Receptor Substrate 2) [62, 63], MMP11 (Matrix Metalloproteinase 11) [64], aquaporin (AQP6) [65], TTC17 (Tetratricopeptide Repeat Domain 17) [66], SEPHS2 (Selenophosphate Synthetase 2) [67], PPP6R2 (Protein Phosphatase 6 Regulatory Subunit 2) [68], PPAPDC1A (Phosphatidic Acid Phosphatase Type 2 Domain Containing 1A) [69], STIM1 (Stromal Interaction Molecule 1) [70], LMOD2 (Leiomodin 2) [71], PPP4C phosphoprotein phosphatase catalytic subunit (PPPCs) [72], RAC1 [73], CCNB2 (Cyclin B2) [74], RAMP1 (Receptor activity modifying protein 1) [75], FOS [76], LINC00899 [77], and phosphatidylcholine (PC) [78] are involved in breast cancer metastasis. These RNAs can serve as an independent and external validation to your computational framework. Future research should explore this RNA panel in tissue and liquid biopsy samples to thoroughly establish their diagnostic, prognostic, and therapeutic values.

## Conclusion

Lymph node metastasis is a critical and influential occurrence in the advancement of breast cancer. To find mRNA targets for lymph node metastasis in breast cancer, we applied an AI-based framework in this study. The suggested method selected the top five mRNAs involved in lymph node metastasis including GDF5, BAHCC1, LCN2, FGF14-AS2, and IDH2. This research has the potential to establish a comprehensive understanding of potential candidate genes that may play a crucial role in the early detection of lymph node metastasis in patients with breast cancer. The differential expression patterns of these genes between lymph node-positive and lymph node-negative tumors, as well as their association with lymph node metastases, highlight their clinical relevance. Further validation and investigation of these genes could lead to the development of more accurate prognostic tools and targeted therapies for breast cancer patients with lymph node involvement.
